# Impact of Strain Variation of *Dichelobacter nodosus* on Disease Severity and Presence in Sheep Flocks in England

**DOI:** 10.3389/fvets.2021.713927

**Published:** 2021-08-16

**Authors:** Emma M. Monaghan, Naomi S. Prosser, Jessica Witt, Katharine E. Lewis, Elizabeth Nabb, Matt J. Keeling, Kevin J. Purdy, Laura E. Green

**Affiliations:** ^1^Institute of Microbiology and Infection, College of Life and Environmental Sciences, University of Birmingham, Birmingham, United Kingdom; ^2^School of Veterinary Medicine and Science, University of Nottingham, Leicestershire, United Kingdom; ^3^School of Life Sciences, University of Warwick, Coventry, United Kingdom; ^4^Zeeman Institute, SBIDER: Systems Biology & Infectious Disease Epidemiology Research, Warwick Mathematics Institute, University of Warwick, Coventry, United Kingdom

**Keywords:** sheep, footrot, *Dichelobacter nodosus*, elimination, strains, persistence

## Abstract

*AprV2* and *aprB2* are variants of the apr gene of *Dichelobacter nodosus*, the cause of footrot in sheep. They are putative markers for severe and mild disease expression. The aim of our study was to investigate the distribution of *aprV2* and *aprB2* in flocks with and without footrot. Our hypotheses were that both strains are present in endemically affected flocks, with *aprB2* and *aprV2* associated with mild and virulent phenotypes respectively but that *D. nodosus* is not present in flocks without footrot. Alternatively, *aprB2* persists in flocks without footrot. Despite extensive searching over 3 years only three flocks of sheep without footrot were identified. *D. nodosus* was not detected in these three flocks. In one further flock, only mild interdigital dermatitis was observed, and only *aprB2* was detected. Twenty-four flocks with endemic footrot of all severities were sampled on three occasions and all were positive for *D. nodosus* and the *aprV2* variant; *aprB2* was detected in only 11 of these flocks. *AprB2* was detected as a co-infection with *aprV2* in the 22% of samples positive for *aprB2* and was more likely in mild footrot phenotypes than severe. *Dichelobacter nodosus* serogroups were not associated with footrot phenotype. We conclude that *D. nodosus*, even *aprB2* strains, do not persist in flocks in the absence of footrot. Our results support the hypothesis that *aprB2* is associated with mild footrot phenotypes. Finally, we conclude that given the small number of flocks without footrot that were identified, footrot is highly endemic in English sheep flocks.

## Introduction

Footrot is an economically important disease of sheep that reduces health, welfare and productivity (1–3). Footrot is caused by *Dichelobacter nodosus* (4–6), a facultative anaerobe that invades damaged interdigital epidermis (7) and has two disease presentations: interdigital dermatitis (ID) where there is inflammation of the interdigital skin and severe footrot (SFR) where the hoof horn separates from the underlying tissue (4, 6). ID and SFR vary in severity of pathology (8) ranging from minimal visible lesion (score 1) to most of the foot affected (score 4). Throughout the manuscript footrot refers to all severities of ID and SFR. *Dichelobacter nodosus* persists on feet with footrot for several months, but only persists on healthy feet for up to 2 weeks (9). *Dichelobacter nodosus* spreads between sheep *via* pasture, surviving off host for a few days in damp conditions (4, 9) but in dry conditions it does not persist off sheep sufficiently long for transmission to occur (9, 10). Due to the epidemiology of *D. nodosus*, Beveridge (4) and recently Clifton et al. (9) proposed that it should be possible to eliminate *D. nodosus* from flocks by eliminating all signs of footrot i.e., by eliminating ID and SFR.

Beveridge's (4) evidence led to a campaign to eliminate footrot from certain states in Australia using a flock by flock elimination program (11, 12). Elimination was successful in some flocks but there were reports that less virulent strains of *D. nodosus* were difficult to eradicate because they persisted in cattle which do not develop disease (13–16).

To manage these less virulent, persistent, strains of *D. nodosus* in New South Wales, Australia, flocks with low prevalence of foot lesions were considered to have eliminated “virulent” footrot and considered footrot free (11). Considerable research effort has since been spent trying to differentiate “benign” and “virulent” strains of *D*. nodosus using biochemical, and more recently genetic techniques. This is summarized in McPherson et al. (17) who concluded that no laboratory technique differentiates *D. nodosus* strains using the legislated flock phenotype definitions of “benign” and “virulent” footrot (17). This indicates that either the clinical definition of benign and virulent is incorrect, or that the methods do not differentiate benign and virulent strains correctly, or both.

Recently, variants of the extracellular protease apr (*aprB2* and *aprV2*) have been proposed to differentiate benign and virulent strains of *D. nodosus* (18). In the field, *aprV2* has been associated with severe footrot (19–21). However, in some cases, *aprB2* has been detected without *aprV2* in flocks with severe footrot (22), and, as stated above, *aprV2*/*B2* did not differentiate benign and virulent footrot at flock level in a cross sectional study in Australia (17).

Footrot has been present in sheep flocks in the UK for centuries (23). It is highly endemic, in 2013 and 2014 farmers reported that >90% of flocks had footrot (ID and SFR) (24). There is no discernable spatial clustering of serogroups of *D. nodosus* in England, indicating homogenous mixing of strains between flocks and emphasizing the highly endemic nature of footrot (25). In the UK, the *aprV2* variant dominates in flocks and feet with previous estimates that <7% of samples positive for *aprV2* contain *aprB2*, (26–28). Since most flocks in England with footrot have both ID and SFR these findings could support the hypothesis that *aprV2* is associated with “virulent” SFR and *aprB2* with “benign” ID, but it does not refute the possibility that, as in Sweden (22), *aprB2* is also associated with SFR.

**Figure 1:**
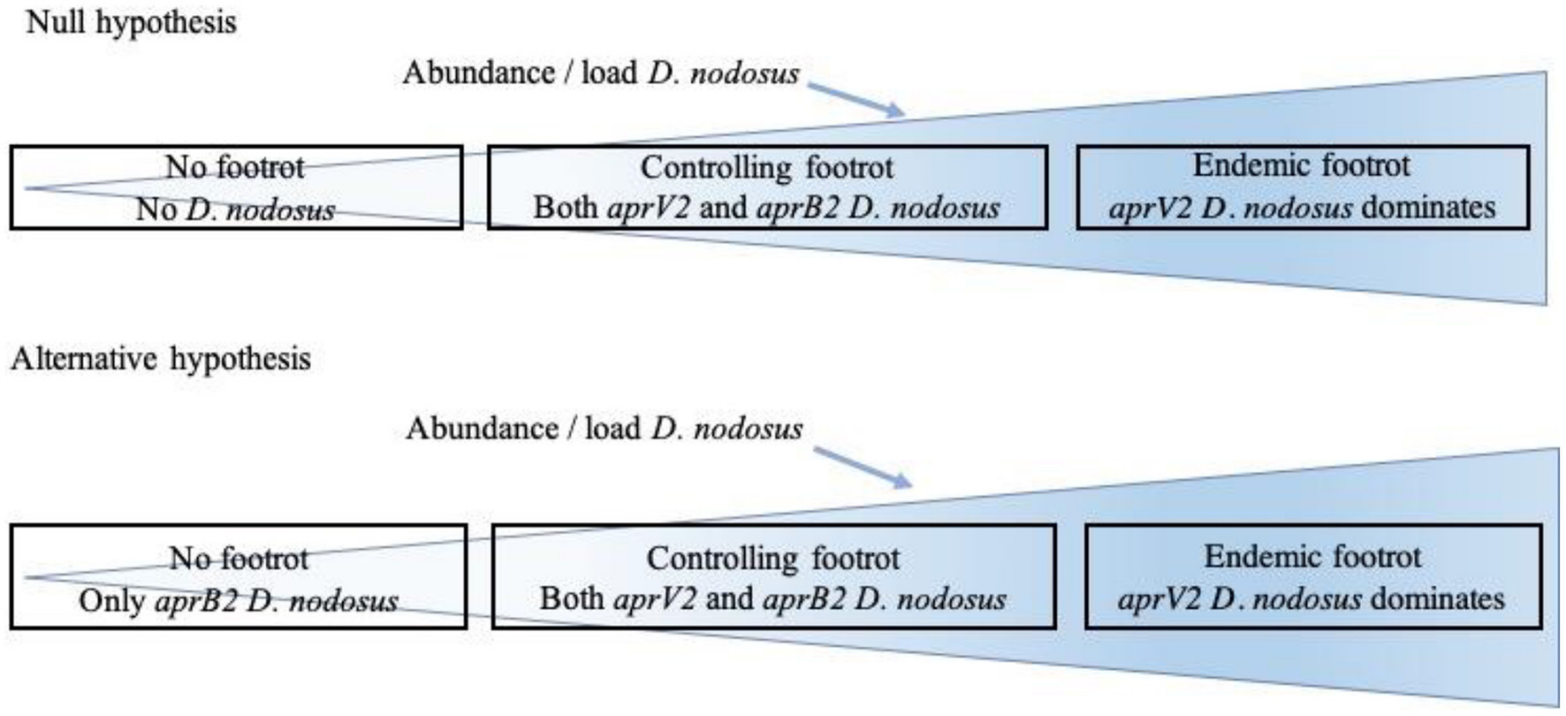
Relationship hypothesized between presence of clinical signs of footrot and presence of *Dichelobacter nodosus, aprV2* and *aprB2*.

The aim of the current study was to investigate the distribution of *D. nodosus* in flocks with and without footrot and the association between *aprV2* and *aprB2*, the presence of disease and disease severity (ID and SFR). We hypothesized that *D. nodosus*, the cause of footrot in sheep, is not present in flocks where there is no footrot (no foot lesions on inspection of all feet) with the alternative hypothesis that *aprB2* strains of *D. nodosus* persist in flocks with no signs of footrot (Figure 1). We also hypothesized that when footrot is endemic in a flock *aprV2* strains of *D. nodosus* dominate over *aprB2* strains. To test these hypotheses, we investigated sheep from 11 flocks where farmers reported no clinical signs of footrot and sheep from 24 flocks with a full range of footrot severities (8).

## Materials and Methods

### Ethical Approval

This study was carried out with approval from the Biomedical and Scientific Research Ethics Committee (BSREC REGO-2014-620) and the Animal Welfare and Ethical Review Committees (AWERB 16/13-14) at the Universities of Warwick and Birmingham, respectively.

### Selection and Data Collection From Flocks Where Farmers Reported No Footrot

In 2013 (24) and 2014 (29) postal questionnaires were sent to 4,000 randomly selected lowland English sheep farms. There were 884 farmers who responded to both questionnaires and 12 of these had reported that they had not seen footrot in either year. The 12 farmers were contacted in 2016, and five reported that they had not seen footrot in their flock for at least 4 years. In a separate snowballing exercise where the goal was to identify all flocks without footrot in England (30), sheep industry experts were asked to identify flocks without footrot and to suggest other experts who might know of flocks with no clinical signs of footrot. This occurred over a period of 3 years and a further six flocks were identified. The owners of these 11 flocks agreed to participate in the study. Two of three trained researchers (EM, EN, NP) visited each flock. In four flocks <15% of sheep were present for examination; these flocks were discarded because it was not possible to examine all sheep and feet in the flock. In the remaining seven flocks (Table 1), initially the locomotion of all sheep in the flock was scored using a validated system (31). When a sheep had a locomotion score > 0 (31) all four feet were examined and scored for footrot severity (8) into ID and SFR with severity scores 1 which are a visible lesion through to 4 more than 50% of the skin/claw affected. Feet were then swabbed with a sterile cotton swab with a downward vertical wipe repeated five times. When fewer than 15 sheep were lame, all four feet of a number of non-lame ewes and rams were also scored and swabbed up to a total of 15 sheep where possible, as for the 24 flocks with footrot (see section Selection and Data Collection From 24 Flocks in England With Clinical Footrot). In four flocks where no lame sheep were observed, all feet of all sheep were examined. In three flocks all feet were swabbed, in the fourth flock lesions were observed on some feet and these were swabbed and in addition, all four feet of every fifth ewe were swabbed. The swabs were placed in amies charcoal and stored at −20°C. A total of 496 foot swabs were collected (Table 1).

**Table 1:** Observation of footrot and detection of *D. nodosus, aprV2* and *aprB2* in seven flocks in England where farmers reported “no footrot” for at least 4 years.

**Flock**	**Years “with no” FR**	**Flock size**	**No. (%) sheep examined**	**No. foot swabs**	**Sheep with FR^**a**^ No (%)**	**Feet with FR^**b**^ No (%)**	**Feet with Dn^**b**^ No. (%)**	**Feet with both FR and Dn^**b**^ No. (%)**	**Feet with *aprV2*^**c**^**	**Feet with *apr B2*^**c**^**
**Lameness/footrot observed at inspection**										
A	5	18	14 (78)	56	1 (7)	3 (5)	22 (39)	3 (14)	20	0
C	5	73	16 (22)	64	8 (50)	15 (23)	8 (13)	6 (75)	8	2
F	29	32	10 (31)	40	1 (10)	2 (5)	1 (3)	0	1	0
K^*^	14	232	232 (100)	48	7 (3)	9 (19)	27 (56)	6 (22)	0	27
**No Lameness/footrot observed at inspection**										
H	25	28	28 (100)	112	0	0	0	0	0	0
I	7	11	11 (100)	44	0	0	0	0	0	0
J	6	21	21 (100)	84	0	0	0	0	0	0

*Alphabetical letter for flock ID is the date order visited; FR, footrot, both interdigital dermatitis (ID) and severe footrot (SFR); No., number; %, percentage; Dn, Dichelobacter nodosus*.

a *Of sheep examined*.

b *Of feet examined*.

c *Of D. nodosus positive feet*.

* *Merino sheep, no lame sheep and footrot severity score <2 observed (8)*.

### Selection of and Data Collection From 24 Flocks in England With Clinical Footrot

An 18-month clinical trial was conducted on 44 flocks with footrot throughout England (32). A total of 24 farmers consented to being in this study, flocks were 100–500 breeding ewes and a prevalence of >5% lameness (Table 2). The flocks were visited on three occasions; autumn 2015 (visit 1), spring 2016 (visit 2) and autumn 2016 (visit 3). At each visit, sheep locomotion was scored as previously described. Up to 15 lame ewes, locomotion score >1 or, when there were fewer than 15 lame ewes, a combination of lame and non-lame ewes, were selected and all four feet were scored for footrot severity and swabbed. Swabs were stored in sterile cryotubes containing 300 μl sterile phosphate buffered saline (PBS) and stored at −20°C. A total of 3,506 foot swabs were collected and those from healthy feet and footrot affected feet (*n* = 2,338) were processed (Supplementary Table 1).

**Table 2:** Summary information on 24 flocks with clinical footrot: flock size, prevalence of lameness by visit, use of Footvax, co-grazing with cattle, detection of *D. nodosus* variant *aprB2* and detection of serogroups of *D. nodosus*.

**Flock**	**Flock size**	**Prevalence of lameness (%)**			Use Footvax^*^	**Co-graze with cattle**	*AprB2* positive^**^	**Serogroups**
		**Visit 1**	**Visit 2**	**Visit 3**				
1	350	6.7	10.0	7.6		✓	✓	A; B; C; D; E; H
2	360	6.5	15.7	9.6		✓	✓	A; B; D; F; H; I
3	200	12.7	4.3	9.4			✓	B; C; H
4	270	10.9	15.0	6.7			✓	B; C; D; E; H; I
5	200	11.6	19.8	4.1	✓	✓	✓	B; C; E; F; H; I
6	350	13.3	7.5	8.4		✓		B; C; D; G; H; I
7	180	5.6	5.9	2.6				B; H; I
8	400	10.6	12.5	4.0		✓	✓	A; B; C; E; H
9	300	9.3	2.4	6.7		✓		A; F; H
10	400	4.2	5.6	6.5				A; B; H
11	500	4.4	4.0	4.0			✓	A; B; C; H
12	320	5.2	8.2	8.2	✓	✓	✓	A; B; C; E; F; H
13	300	5.3	12.7	3.8		✓	✓	B; C; G; H
14	200	7.8	4.4	6.4	✓			B; G; H
15	340	7.6	2.7	3.3	✓	✓	✓	A; B; D; E; G; H
16	220	9.1	10.0	7.4		✓		A; B; C; D; E; H
17	360	10.4	5.3	10.8	✓			A; B; C; D; H; I
18	300	5.7	6.7	6.0	✓		✓	A; B; C; D; E; H; I
19	400	9.7	7.7	10.0		✓		A; B; D; E; H; I
20	330	9.6	12.5	7.0				A; B; H
21	200	8.7	5.3	6.7		✓		A; B; C; F; H
22	260	9.5	9.6	7.5				0
23	113	18.9	9.4	9.1				A; H
24	350	9.7	11.3	12.5	✓			A; B; H

* *Footvax ™ (MSD Animal Health) is the commercial vaccine licensed for footrot in sheep*.

** *All flocks were aprV2 D. nodosus positive*.

### Laboratory Processing of Foot Swabs

Swabs were centrifuged at 1,600 g for 8 min. Swabs from the putative footrot-free farms were processed as individual samples and were not pooled prior to DNA extraction. Supernatant from swabs taken from the 24 endemically affected flocks were pooled by visit to a flock into 10 phenotypes: healthy feet from sheep where all four feet were healthy (AH), healthy feet from sheep where one or more feet had any severity of footrot (HD), and separate pools for feet with ID by severity score 1, 2, 3, or 4 and SFR by severity score 1, 2, 3, or 4 (8). There were a total of 395 pooled samples (Supplementary Table 1); 309 (78.2%) with 2–32 swabs per sample and 86 (21.8%) single swab samples. There were 12–23 pooled samples per flock (Table 3). The supernatant was removed after pooling and centrifuging the swabs and the pellets re-suspended in 500 μl of PBS. DNA was extracted as described previously (33), eluted into 45 μl aliquots and stored at −20°C.

**Table 3:** Number and percentage of samples collected and positive for *Dichelobacter nodosus, aprV2* only and both *aprV2* and *aprB2* for 24 flocks in England with clinical footrot.

Flock ID^**^	No. samples^*^		***D. nodosus***		***aprV2*** **only**		***aprV2*** **and** ***aprB2***	
	**No**.	**%**	**No**.	**%**	**No**.	**%**	**No**.	**%**
4	12	3.0	12	100	8	66.7	4	33.3
19	20	5.1	20	100	20	100	0	0.0
8	22	5.6	21	95.5	5	23.8	16	76.2
20	20	5.1	19	95.0	19	100	0	0.0
2	14	3.5	13	92.9	12	92.3	1	7.7
13	14	3.5	13	92.9	6	46.2	7	53.8
16	21	5.3	19	90.5	19	100	0	0.0
15	17	4.3	15	88.2	14	93.3	1	6.7
17	15	3.8	13	86.7	13	100	0	0.0
23	19	4.8	16	84.2	16	100	0	0.0
14	12	3.0	10	83.3	10	100	0	0.0
21	23	5.8	19	82.6	19	100	0	0.0
18	14	3.5	11	78.6	2	18.2	9	81.9
5	12	3.0	9	75.0	1	11.1	8	88.9
10	14	3.5	10	71.4	10	100	0	0.0
6	17	4.3	12	70.6	12	100	0	0.0
11	16	4.1	11	68.8	10	90.9	1	9.1
9	18	4.6	11	62.2	11	100	0	0.0
7	12	3.0	7	58.3	7	100	0	0.0
1	19	4.8	11	57.9	6	54.5	5	45.5
3	13	3.3	7	53.8	1	14.3	5	71.4^***^
24	15	3.8	8	53.3	8	100	0	0.0
12	22	5.6	10	45.5	7	70.0	3	30.0
22	14	3.5	1	7.2	1	100	0	0.0
Total	395		298		237		60	

* *1–32 swabs pooled per sample, 86 single swab samples. No., number, D. nodosus, Dichelobacter nodosus*.

** *Flocks are ordered by percentage of samples positive for D. nodosus*.

*** *One sample from this flock was aprB2 positive only*.

#### Detection and Quantification of *Dichelobacter nodosus* and Protease Genes AprV2 and AprB2

*Dichelobacter nodosus* was detected by targeting a 61 bp sequence in the *rpoD* gene, and quantified by qPCR as described previously (6, 34). Samples that were positive for *D. nodosus* were tested for *aprV2* and *aprB2* using the qPCR method of Frosth et al. (22) with a modification made to the probe labeling (*aprV2* probe 6FAM-BHQ1, *aprB2* probe TxRd-BHQ2). The initial denaturation step was 15 min following the Klearkall master mix (LGC Group) methods. Each reaction consisted of 0.4 μM of each primer, 0.1 μM probe, 7.5 μl Klearkall master mix (LGC Group), 0.1 mg/ml^−1^ bovine serum albumin (BSA) solution, 4.85 μl nuclease-free water (Applied Biosystems) and 1 μl template DNA. The DNA did not amplify on 28 occasions and so the process was repeated with 2 μl DNA, a further eight samples amplified.

#### Detection of *Dichelobacter nodosus* Serogroups

Presence of *D. nodosus* serogroups A-I was investigated using the single serogroup target PCR protocol described in Dhungyel et al. (35). Serogroup M was not investigated because there is no serogroup M specific protocol (36). Each reaction consisted of 0.5 μM primer, 2x MyTaq Red mix (Bioline), 0.5 mg/ml^−1^ BSA, 16.5 μl nuclease-free water (Applied Biosystems) and 1μl template DNA. PCR products were visualized using 3% agarose stained with ethidium bromide under ultraviolet light.

### Data Storage and Analysis

All data were stored in Microsoft Excel. The prevalence of lameness (defined as sheep with a locomotion score > 0) and the prevalence of footrot (sheep with any score > 0 on any foot) and the frequency of footrot lesion severity scores (28) were calculated for each flock-visit. The percentage of sheep sampled, swabs/pooled samples positive for *D. nodosus, aprV2*, and *aprB2*, and for serogroups A-I were calculated.

A binomial multivariable mixed effect model was used to investigate associations between detection of *aprV2* and *aprB2* in pooled samples and the explanatory variables footrot disease severity score and serogroup. The outcome was pooled sample with *aprV2* vs. *aprV2* and a*prB2*.

The equation took the form:

$$\begin{array}{c} logit\left({aprV2\;and\;aprB2\;:\;aprV2}_{ijk}\right)\;\sim \;\beta_{0ijk}+\;{\Sigma \beta }_{ijk}X\\ +\;{\Sigma \beta }_{jk}X+e_{k}+e_{j} \end{array}$$

Where *logit (aprV2 and aprB2 : aprV2)* is the outcome variable, β_0_ is the intercept and β_*ijk*_ are a series of explanatory variables varying at *i* (sample), *j* (visit), and *k* (flock) with e the residual error at levels k and j, the residual error at “*i*” was assumed to follow a binomial error distribution. The model was built in a forward-stepwise process (37), using *lme4* (38) in RStudio version 3.6.0 (39). The reference category for footrot severity was ID score 1 because there were few *D. nodosus* positive samples for AH and HD pools and so ID1 provided a stable baseline category. All severity scores were included and serogroups were added when the *p*-value from a likelihood ratio test comparing the model with and without the variable was ≤0.05, when *p* > 0.05 from the likelihood ratio test, the explanatory variable did not improve the model fit and were omitted.

Similarly, three multivariable mixed effect regression models were run using *lme4* (38) with three outcomes: the log [mean (qPCR replicate) +1] for all *D. nodosus*, for *aprV2* only and for *aprV2* + *aprB2*. An explanatory variable of *aprV2* load was forced into the *aprV2* + *aprB2* model to adjust for *aprV2* load in the sample and remove confounding *aprV2* load with explanatory variables. The explanatory variables were disease phenotype, baseline ID1, as for the binomial models and serogroup and random variables for visit (j) and flock (k), with sample (i) fitted as the unit of observation in a three-level model (37).

The equation took the form:

$$\begin{array}{c} \log\left(qPCR_{ijk}\right)=\beta_{0ijk}+\;{\Sigma \beta }_{ijk}X+\;{\Sigma \beta }_{jk}X+e_{k}+e_{j}+e_{i} \end{array}$$

Where *log(qPCR)* is the log_10_ [mean (qPCR replicate load)] of the outcome variables (all *D. nodosus, aprV2, aprV2*, and *aprB2*), β_0_ is the intercept and β_*ijk*_*X* are a series of exploratory variables varying at *i* (sample), *j* (visit), and *k* (flock) with *e*, the residual error at sample level. Confidence intervals and Wald's *p*-values were obtained from *broom.mixed* (40).

To investigate whether pooling samples reduced the detection of serogroups, the data were simulated assuming that the probability of detecting a particular serogroup for a given sample was dependent on both lesion severity score and flock of origin:

$$\begin{array}{cc} Prob\;\left(detection\;of\;a\;serogroup\;X\;|\;lesion\;severity,\;Flock\right)\\ = & D_{Severity}\;\times \;P_{Flock,}\left(X\right) \end{array}$$

Where the disease state (*D*) and flock-level prevalence of lesion (*P*) were determined such that the sample results are recovered. This was done using reversible jump Markov Chain Monte Carlo (RJMCMC) (41) using the likelihood of obtaining the pooled results from the independent samples of feet within each pool. The mean and 95% credible intervals for each parameter were calculated. The model assumed independence between serogroups. The model's performance was assessed by comparing the observed results from 2,338 samples across the 24 flocks with footrot with the predicted footrot severity scores.

## Results

### Flocks in England Where Farmers Reported No Footrot

**Figure 2:**
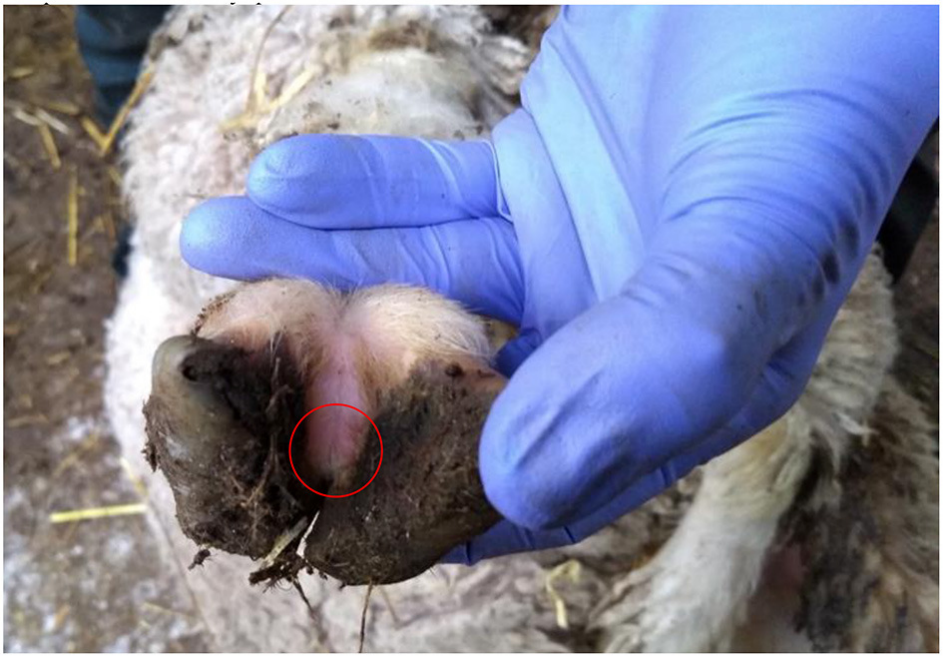
Photograph of a mild interdigital lesion (whitish skin in the circle) from flock K where no sheep were lame and only *aprB2* variant of *D. nodosus* was detected.

There were 7 flocks where farmers thought footrot was absent and where all sheep were inspected. In three flocks (H, I, and J) footrot and *D. nodosus* were not detected (Table 1). In three further flocks (A, C, and F) sheep were lame, footrot of all severities was observed, and *D. nodosus* and *aprV2* were detected (Table 1); *aprB2* was also detected in flock C. In flock K, a flock of 232 Merino sheep, no sheep were lame but mild interdigital lesions were observed in about 3% of sheep, although there was no necrotic smell typical of footrot (Figure 2). Only the *aprB2* variant and not the *aprV2* variant of *D. nodosus* was detected.

### Twenty-Four Flocks With Clinical Footrot

**Figure 3:**
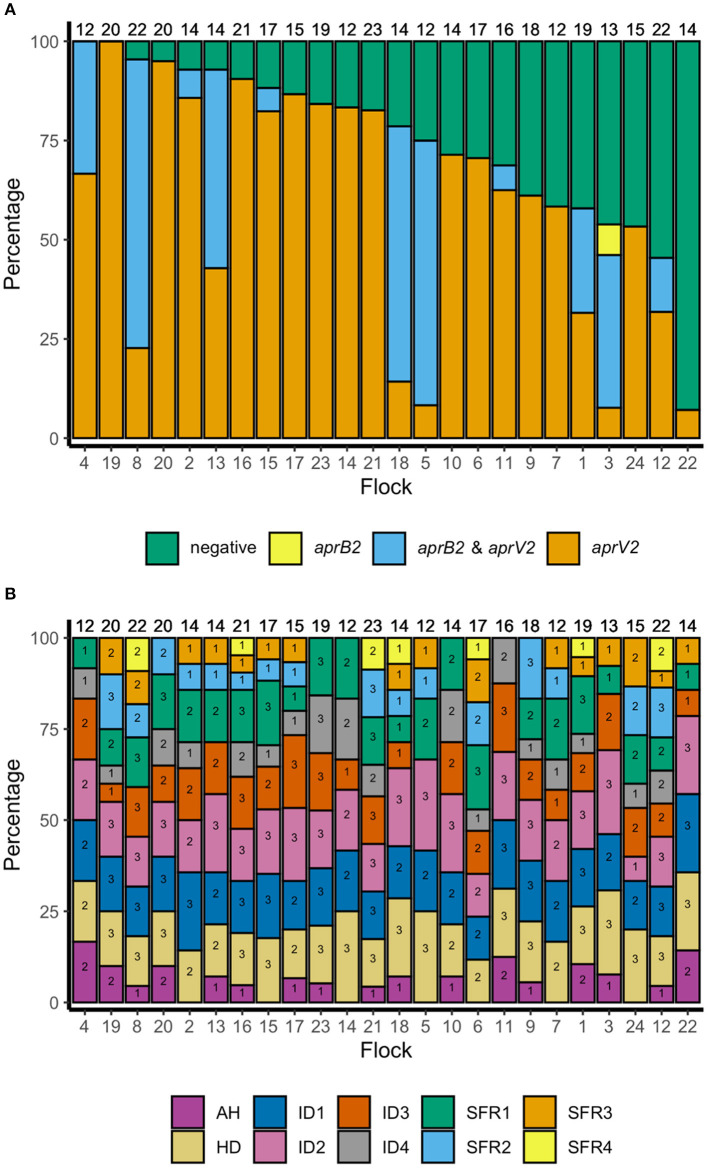
**(A)** Percentage of samples negative for *D. nodosus* and positive for *D. nodosus* by *aprV2* only, *aprB2* and *aprV2* and *aprB2* only in descending order of aprV2 only: 395 pooled samples from 24 flocks in England. Number at top of each bar = number of pooled samples over three visits, number at the bottom = flock identification. **(B)** Percentage of samples positive by foot phenotype: 395 pooled samples from 24 flocks in England. Top of column number = total number of pooled samples per flock for all three visits. Number within each bar = number of pooled samples per flock for all three visits by foot phenotype. AH, all healthy feet from sheep where all four feet were healthy; HD, Healthy feet from sheep where one or more feet had signs of footrot; ID, Interdigital dermatitis scores 1–4; SFR, Severe footrot scores 1–4. Number at base of bar = flock identification number, ordered as for **(A)**.

**Figure 4:**
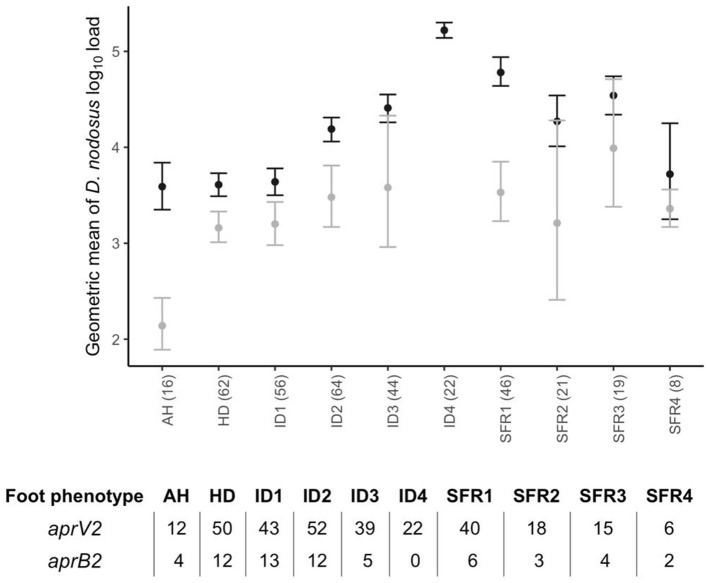
Geometric mean and 2 standard errors for log_10_ *aprV2 D. nodosus* load (black) and log_10_ *aprB2 D. nodosus* load (grey) by foot phenotype from 395 samples from 24 flocks in England with clinical footrot. AH, all healthy feet from sheep where all four feet were healthy; HD, Healthy feet from sheep where one or more feet had signs of footrot; ID, Interdigital dermatitis scores 1–4; SFR, Severe footrot scores 1–4. Number in brackets after each foot phenotype on the x-axis is the total number of pooled swabs positive for *aprV2* or *aprB2* in each foot phenotype category. The associated table shows the split between samples *aprV2* or *aprB2* positive out of total number positive.

**Figure 5:**
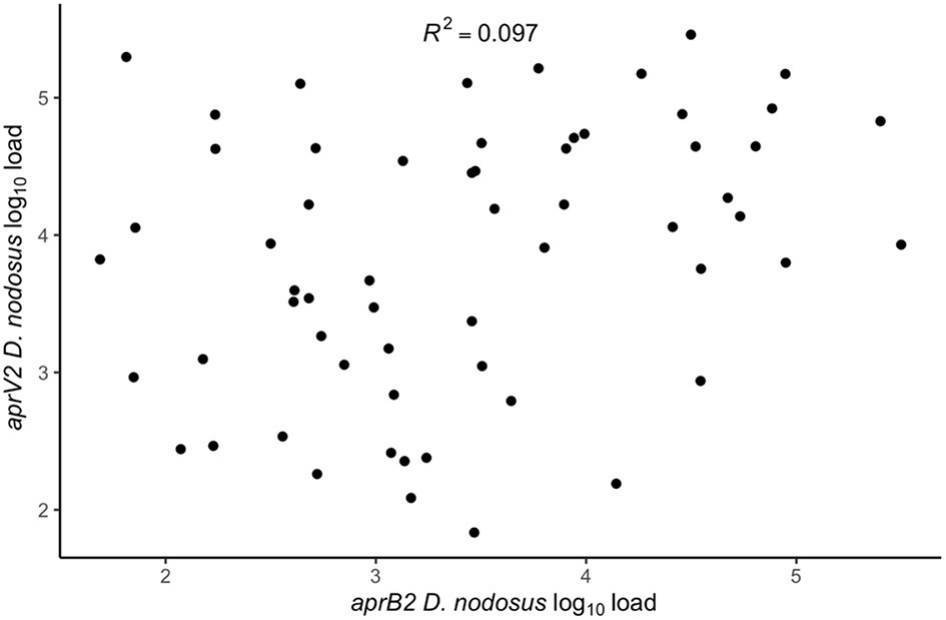
Within swab association between *aprV2 D. nodosus* log_10_ load and *aprB2 D. nodosus* log_10_ load from 60 samples positive for both *aprV2* and *aprB2 D. nodosus* from 3 visits (1–3) to 24 flocks in England with clinical footrot. The correlation coefficient (*R*^2^) was calculated using the built-in lm function in R Studio Version 3.6.0 (39) to calculate a simple linear regression. *P*-value = 0.02.

There were lame sheep in all 24 flocks with footrot. Footrot lesions, *D. nodosus*, and *aprV2* were detected in all 24 flocks (Figures 3A,B). There were 298 (75.4%) *D. nodosus* positive samples (Table 3). *AprV2* was detected in all 24 flocks and 297 of the 298 *D. nodosus* positive samples (Table 3) and *aprB2* was detected in 11 flocks and 61 (22%) samples; *aprB2* was detected without *aprV2* in only one sample pooled from 2 swabs with severity score SFR 3, *aprV2* was detected in this flock-visit (Figure 3A). The geometric mean load of *aprV2* was typically greater than the load of *aprB2* in all foot phenotypes (Figure 4) but there was a positive correlation (*r* = 0.097) between the log load of *aprV2* and *aprB2* (Figure 5).

There were 231/298 (77.5%) *D. nodosus* positive samples where at least one serogroup was detected. All nine serogroups (A-I) were detected in 63 combinations with a median of 5 (IQR 3–6) serogroups per flock. Serogroups H, B, and A were most frequently detected, in 38.6, 30.2, and 20.1% of samples, respectively (Supplementary Tables 2–4). In the 67 *D. nodosus* positive samples where serogroups were not detected, the qPCR load was below the limit of detection for serogroup PCR in 60 samples and the remaining 7 had sufficient *D. nodosus* load but no serogroup was detected; it is possible these were serogroup M.

In the multivariable mixed effect binomial regression model, feet with ID 3–4, or SFR of any severity score were more likely to have *aprV2* only than both *aprV2* and *aprB2*, compared with ID 1, indicating that presence of *aprB2* was associated with lower footrot severity scores. Serogroups C and E were less likely to be present in samples with *aprV2* alone than *aprV2* and *aprB2* (Table 4), other serogroups were not significantly associated with *apr* variant.

**Table 4:** Binomial multivariable mixed effect regression model of pooled samples that were *aprV2* only vs. *aprV2* and *aprB2* gene variants by footrot phenotype and serogroup in 298 samples from three visits to 24 sheep flocks in England.

	**No**.	**%**	**No. *aprB2* positive**	**% *aprB2* positive**	**OR**	**LCI**	**UCI**
Intercept					–		
**Sample classification**							
ID score 1	43	14.4	13	30.2	Ref		
AH	12	4.0	4	33.3	7.23	0.21	247.66
HD	50	16.8	12	24.0	0.57	0.07	5.00
ID score 2	52	17.4	12	23.1	0.14	0.02	1.38
ID scores 3–4^*^	61	20.5	5	8.2	**0.01**	** <0.01**	**0.15**
SFR score 1	40	13.4	6	15.0	**0.02**	** <0.01**	**0.44**
SFR scores 2–4^*^	40	13.4	9	22.5	**0.01**	** <0.01**	**0.34**
**Number of samples pooled (range x-y)**							
1 unit increase	298	100			**0.81**	**0.66**	**1.00**
**Serogroup E**							
No	257	86.24	43	16.7			
Yes	41	13.76	18	43.9	**11.19**	**0.99**	**126.27**
**Serogroup C**							
No	261	87.58	39	14.9			
Yes	37	12.42	22	59.5	**19.80**	**2.00**	**195.83**

*Flock variance 28.5, visit variance 17.1, sample variance constrained to a binomial distribution. No., number, %, percentage; OR, odds ratio; and OR >1 indicates a sample was more likely to have aprB2 strain variant, an OR < 1 indicates a samples was less likely to have the aprB2 variant; LCI, lower 95% confidence interval of OR; UCI, upper 95% confidence interval of OR; Bold, variable significant at P ≤ 0.05 Wald's-test, ID, interdigital dermatitis; AH, all healthy feet from sheep where all four feet were healthy; HD, Healthy feet from sheep where one or more feet had signs of footrot; ID, Interdigital dermatitis; SFR, Severe footrot*.

* *Severity scores pooled when a group had <10 observations*.

In the mixed effect multivariable regression models, the load of *D. nodosus* and *aprV2* increased with severity of ID score compared with ID 1 and then plateaued once feet had SFR (Table 5; Figure 4). Load of *aprB2*, after adjusting for load of *aprV2*, was higher in feet with ID score >1 than ID 1, but did not increase with disease severity as observed for *aprV2*. This is also illustrated in Figure 4. A higher load of all *D. nodosus* was significantly associated with the six most prevalent serogroups (A, B, C, E, F, H), a higher load of *aprV2* was associated with serogroups A, B, E, and H. An increasing load of *aprB2* was associated with serogroup F. When serogroup positive samples from single swabs only (*n* = 53) were analyzed, the patterns of association were similar to those for pooled samples but confidence intervals were wider because of the reduced power of the dataset (data not shown). The simulated data indicated that pooling samples did not reduce the detection of the number of serogroups per sample (Supplementary Table 5).

**Table 5:** Three multivariable mixed effects regression models of pooled samples by load of all *D. nodosus*, only *aprV2*, and *aprB2* adjusted for *aprV2* from 3 visits to 24 sheep flocks in England with clinical footrot.

	**All** ***D. nodosus*** **(** ***n*** **=** **298)**			***aprV2*** **only (** ***n*** **=** **236)**			***aprB2*** **and** ***aprV2*** **(** ***n*** **=** **62)**		
**Variables**	**Coef**	**LCI**	**UCI**	**Coef**	**LCI**	**UCI**	**Coef**	**LCI**	**UCI**
(Intercept)	3.33	3.10	3.55	3.45	3.20	3.70	1.81	0.95	2.67
ID1	Ref			Ref			Ref		
AH	−0.22	−0.62	0.19	−0.39	−0.87	0.09	**−0.95**	**−1.86**	**−0.05**
HD	−0.11	−0.36	0.14	−0.12	−0.42	0.17	−0.07	−0.61	0.48
ID2	**0.32**	**0.06**	**0.57**	0.20	−0.09	0.49	0.11	−0.46	0.68
ID score 3–4	**0.85**	**0.60**	**1.10**	**0.77**	**0.50**	**1.05**	0.57	−0.16	1.30
SFR score 1	**1.02**	**0.75**	**1.29**	**1.00**	**0.69**	**1.30**	0.28	−0.49	1.04
SFR score 2–4	**0.68**	**0.41**	**0.96**	**0.67**	**0.35**	**0.98**	0.61	−0.01	1.23
Serogroup A	**0.35**	**0.16**	**0.54**	**0.30**	**0.10**	**0.51**	0.05	−0.61	0.72
Serogroup B	**0.31**	**0.14**	**0.48**	**0.34**	**0.16**	**0.53**	−0.01	−0.59	0.57
Serogroup C	**0.24**	**0.00**	**0.48**	0.15	−0.14	0.45	0.14	−0.39	0.67
Serogroup D	0.10	−0.16	0.37	0.13	−0.16	0.42	0.09	−0.62	0.80
Serogroup E	**0.46**	**0.20**	**0.72**	**0.46**	**0.12**	**0.81**	0.40	−0.21	1.02
Serogroup F	**0.77**	**0.19**	**1.35**	0.58	−0.05	1.21	**1.88**	**0.15**	**3.61**
Serogroup G	0.38	−0.07	0.83	0.20	−0.24	0.65	–	–	–
Serogroup H	**0.46**	**0.30**	**0.62**	**0.36**	**0.18**	**0.55**	0.06	−0.41	0.53
Serogroup I	0.11	−0.19	0.41	0.17	−0.16	0.50	−0.09	−0.99	0.81
Log load *aprV2*	–		–			**0.33**	**0.08**	**0.58**	
**Random effects**									
Farm	0.21			0.17			0.12		
Visit	0.18			0.21			0.53		
Residual	0.61			0.59			0.62		

*Coef, coefficient; LCI, lower 95% confidence interval of the coefficient; UCI, upper 95% confidence interval of the coefficient. No observations with serogroup G in the 62 samples with aprB2, AH, healthy foot from sheep with 4 healthy feet; HD, healthy foot from sheep with at least one foot with ID/SFR > score 0; ID, interdigital dermatitis; SFR, severe footrot; scores 1–4, severity scores (8). Explanatory variables significant at P ≤ 0.05 (Wald's-test) are highlighted in bold*.

*Scores pooled when <10 samples in a severity score.

## Discussion

Our study supports previous findings that *D. nodosus* and footrot are highly endemic in flocks in England, with only three flocks without footrot detected over 4 years. The results from the current study, together with published work from other countries confirm our hypothesis that *D. nodosus*, the causative agent of footrot in sheep, is present in flocks where there is footrot and is not present where there is no footrot. As proposed by Beveridge (4) and Clifton et al. (9), *D. nodosus* is absent when there are no sheep with footrot. In the seven flocks that were examined that were putatively free from footrot, three flocks that had footrot 6–20 years previously had no footrot lesions and no *D. nodosus*, four flocks had sheep with lesions, and *D. nodosus* was detected in all four flocks, even flock F where only one sheep had footrot lesions. *Dichelobacter nodosus* was detected in all 24 endemically affected flocks. These findings do not contradict other published work as discussed below but they do add clarity to a previously confusing narrative.

One important protocol in the examination of putatively footrot-free flocks was that every foot of every sheep in the flock was examined until footrot was observed or all feet had been examined. Because mild lesions do not always cause lameness (42) it is not possible to rely on detection of lameness alone and is essential to inspect all feet to be certain that footrot was absent from a flock. Similarly, using “freedom from disease” sampling and not examining all feet would have compromised our interpretation, flock F exemplifies how this would have produced doubt into our results. A second important methodological design was the use of a strict definition of no footrot that was just that, no foot with a footrot severity score > 0, a definition that is consistent for all scoring systems across the globe (8, 43–46) but not used in many studies.

Our definition of footrot-free was stricter than that used in other studies that have suggested *aprB2* can persist in flocks without footrot. In those studies not all feet were inspected and a severity score of 1 was considered negative for footrot. Studies include those from Germany (47), Australia (48–52), Switzerland (53, 54), and Norway (55). Our results from flock K and the 24 endemically affected flocks highlight the challenge of studying flocks with footrot to elucidate the relationship between strain variation and disease severity because once infected with *D. nodosus*, disease severity varies over time and because feet will be contaminated between sheep in the same group, elucidating the role of strain variation on disease severity is difficult.

It was extremely challenging to identify even three flocks free from all clinical signs of footrot in England. The three flocks free from footrot were small (<50 ewes) and none were from the previous study of 1,400 flocks (24, 29) where ~80% of flocks had >200 ewes. It seems intuitive that footrot is more likely to be absent in small flocks than larger flocks because they may be below the critical community size for persistence of footrot and so natural fade out of disease can occur (56). Indeed, three farmers reported no signs of footrot for 6–20 years but none had actively eliminated footrot, although active elimination of footrot, through inspection, rapid treatment and culling would require less effort in small flocks than large. Two of the three flocks were also biosecure (strict control of sheep entering the farm) and therefore more likely to prevent footrot from being reintroduced through purchase of sheep or sheep straying onto the units. There were 35,500 sheep holdings in England in 2018 (57). Surprisingly, 36% had <50 ewes (57) so it might be possible that there are more flocks without footrot in England, although our snowballing exercise did not identify them, and many of the sheep specialists involved could not recall any flocks free from footrot (personal communication). Our results further support the highly endemic nature of *D. nodosus* and footrot in sheep in England as highlighted in Prosser et al. (25) who reported that serogroups of *D. nodosus* are randomly distributed across England.

We hypothesized that when clinical footrot was endemic in a flock, *aprV2* strains of *D. nodosus* dominate over *aprB2*. This was the case at a flock level in our endemically affected flocks, *aprV2* was present in all 24 flocks with footrot whilst *aprB2* was present in only 11 of these flocks. It was also the case that *aprV2* dominated the samples from the feet with footrot, with *aprB2* only present in 22% of samples. Finally, *aprB2* was less likely to be present in more severe disease phenotypes than *aprV2* (Table 4), with both phenotypes present in less severe phenotypes.

We hypothesized that *Dichelobacter nodosus*, in particular *aprB2*, would persist in flocks without footrot. We did not identify any such flocks and conclude that this hypothesis is false in reference to the flocks we investigated. There was one flock (K) of ~240 Merino sheep, where *aprB2* was detected in the absence of *aprV2* in the current study and we believe this is the first such flock detected in England to date. Flock K was unusual because Merino flocks are rare in the UK. The flock has been in the UK since 1970 and was crossbred with native UK breeds in an attempt to improve wool quality, and so had mixed with native sheep. The current owner treated the whole flock with antibiotics when purchased in 2006 and had not observed footrot since then, and they believed there was no footrot in the flock, they also returned the flock to near pure Merino. The presence of the *aprB2* variant only and mild footrot lesions would fit with the proposal that *aprB2* strains of *D. nodosus* are only capable of causing mild disease (18). However, the management of this flock would also influence the expression of footrot. The owner houses the sheep in straw bedded barns during inclement weather, including heavy rainfall, to protect the valuable fleece. This has the incidental effect of keeping feet dry, which protects from disease expression (9). The owner maintains high levels of biosecurity and the sheep are isolated from all other livestock preventing the introduction of other strains of *D. nodosus*.

Whilst we identified flocks without footrot and where *aprV2* and *aprB2* were absent, apparently after footrot had been in the flocks in the past, we do not know whether *aprV2* and *aprB2* strains can be eliminated from flocks with footrot. Efforts to eliminate footrot in flocks in other countries have focused on elimination of “virulent footrot” which suggests that elimination of all lesions is very difficult in many countries (47–55). From our study we conclude that *aprV2* dominates in flocks where footrot is endemic, and that there is evidence that *aprB2* is associated with less severe footrot. Considering these new findings hypotheses for future study would be:

1. It is easier to eliminate footrot and *D. nodosus* from flocks with only *aprV2*.
2. Where *aprV2* and *aprB2* are present in a flock, *aprB2* will persist longer in elimination programmes.
3. It is not possible to eliminate *aprB2* strains of *D. nodosus*.

There was no variation in disease expression by serogroup, this is consistent with reports that disease severity is not linked to serogroup (5). The current study would support the lack of association between serogroup and disease severity through epidemiological rather than microbiological study. Unlike Prosser et al. (25), pooling samples did not reduce detection of serogroups per flock, probably because more sheep were swabbed per flock-visit in the current study. We did find an association between presence of serogroups C and E and presence of *aprB2* in a foot (Table 4). This might indicate that these serogroups were more likely to be *aprB2* strains of *D. nodosus*, but since *D. nodosus* can seroconvert (58, 59), serogroup is unlikely to be a reliable marker of virulence.

## Conclusion

We conclude that *D. nodosus* did not persist in the flocks without signs of footrot in this study but that these flocks were rare. In all flocks where we saw clinical signs of footrot, *D. nodosus* was present, including three flocks that farmers thought were free from footrot. There is no evidence from our study that *D. nodosus* persists when footrot is not observed in a flock for several years. The relationship between *aprV2* and *aprB2* strains in footrot in endemically affected flocks suggest that *aprB2* is associated with milder footrot lesions. There remain questions on the feasibility of eliminating all *D. nodosus* from endemically diseased flocks, and whether *aprB2* strains would persist for longer than *aprV2* strains of *D. nodosus* in an elimination programme.

## Data Availability Statement

The raw data supporting the conclusions of this article will be made available by the authors, without undue reservation.

## Ethics Statement

The animal study was reviewed and approved by the Biomedical and Scientific Research Ethics Committee University of Warwick (BSREC REGO-2014-620) and the Animal Welfare and Ethical Review Committee (AWERB 16/13-14) University of Birmingham. Written informed consent was obtained from the owners for the participation of their animals in this study.

## Author Contributions

LG and KP designed the study. JW collected samples from the 24 flocks with clinical footrot with assistance from NP and EM. EM, NP, and EN collected samples from putative footrot free flocks. EN assisted in identifying footrot free flocks. EM conducted all laboratory work and statistical analysis with input from LG, KP, MK, and KL. EM and LG wrote the manuscript. All authors contributed to manuscript editing and approved the final manuscript.

## Conflict of Interest

The authors declare that the research was conducted in the absence of any commercial or financial relationships that could be construed as a potential conflict of interest.

## Publisher's Note

All claims expressed in this article are solely those of the authors and do not necessarily represent those of their affiliated organizations, or those of the publisher, the editors and the reviewers. Any product that may be evaluated in this article, or claim that may be made by its manufacturer, is not guaranteed or endorsed by the publisher.
