# Supplementary material for: Impact of Strain Variation of Dichelobacter nodosus on Disease Severity and Presence in Sheep Flocks in England
Source: Front Vet Sci. 2021 Aug 16;8:713927. doi: 10.3389/fvets.2021.713927 (PMC8415419; doi:10.3389/fvets.2021.713927)
Supplement: Supplementary file 1 [file Table_1.DOCX]

Supplementary Table 1. Number of foot swabs collected (2338) and then pooled (395) into ten foot phenotypes for three visits to 24 flocks in England with clinical footrot.

| Flock number | Foot phenotype | | | | | | | | | | No. swabs  per flock ^a^ | No. pooled samples  per flock ^b^ |
| --- | --- | --- | --- | --- | --- | --- | --- | --- | --- | --- | --- | --- |
|  | AH | HD | ID1 | ID2 | ID3 | ID4 | SFR1 | SFR2 | SFR3 | SFR4 |  |  |
| 1* | 8 | 48 | 30 | 21 | 4 | 3 | 7 | 0 | 2 | 1 | 124 | 19 |
| 2* | 0 | 17 | 6 | 5 | 3 | 1 | 5 | 1 | 4 | 0 | 42 | 14 |
| 3* | 4 | 14 | 14 | 9 | 3 | 0 | 4 | 0 | 2 | 0 | 50 | 13 |
| 4* | 12 | 26 | 15 | 10 | 4 | 6 | 1 | 0 | 0 | 0 | 74 | 12 |
| 5* | 0 | 19 | 8 | 8 | 0 | 0 | 3 | 2 | 1 | 0 | 41 | 12 |
| 6 | 0 | 13 | 9 | 12 | 8 | 4 | 8 | 4 | 2 | 1 | 61 | 17 |
| 7 | 0 | 39 | 27 | 18 | 1 | 2 | 3 | 2 | 1 | 0 | 93 | 12 |
| 8* | 16 | 51 | 24 | 18 | 10 | 0 | 11 | 6 | 3 | 4 | 143 | 22 |
| 9 | 16 | 40 | 30 | 17 | 3 | 1 | 5 | 5 | 0 | 0 | 117 | 18 |
| 10 | 28 | 27 | 3 | 7 | 10 | 6 | 2 | 0 | 0 | 0 | 83 | 14 |
| 11* | 20 | 50 | 30 | 17 | 14 | 6 | 0 | 0 | 0 | 0 | 137 | 16 |
| 12* | 4 | 21 | 20 | 10 | 6 | 2 | 3 | 6 | 1 | 5 | 78 | 22 |
| 13* | 4 | 14 | 9 | 9 | 3 | 0 | 2 | 1 | 1 | 0 | 43 | 14 |
| 14 | 0 | 29 | 13 | 6 | 3 | 4 | 4 | 0 | 0 | 0 | 59 | 12 |
| 15* | 0 | 17 | 35 | 41 | 6 | 1 | 6 | 2 | 1 | 0 | 109 | 17 |
| 16 | 4 | 39 | 38 | 33 | 12 | 3 | 16 | 1 | 2 | 2 | 150 | 21 |
| 17 | 4 | 36 | 19 | 40 | 9 | 5 | 4 | 1 | 1 | 0 | 119 | 15 |
| 18* | 12 | 43 | 37 | 8 | 1 | 0 | 3 | 2 | 1 | 1 | 108 | 14 |
| 19 | 24 | 35 | 16 | 20 | 4 | 1 | 4 | 8 | 3 | 0 | 115 | 20 |
| 20 | 8 | 32 | 25 | 40 | 18 | 13 | 6 | 3 | 0 | 0 | 145 | 20 |
| 21 | 4 | 59 | 22 | 13 | 11 | 2 | 13 | 5 | 0 | 7 | 136 | 23 |
| 22 | 8 | 57 | 53 | 10 | 1 | 0 | 1 | 0 | 3 | 0 | 133 | 14 |
| 23 | 16 | 19 | 23 | 25 | 22 | 12 | 10 | 0 | 0 | 0 | 127 | 19 |
| 24 | 0 | 18 | 6 | 6 | 12 | 2 | 3 | 2 | 2 | 0 | 51 | 15 |
| No. swabs ^c^ | 192 | 763 | 512 | 403 | 168 | 74 | 124 | 51 | 30 | 21 | 2338 |  |
| No. pooled ^d^ | 23 | 65 | 61 | 65 | 47 | 27 | 49 | 28 | 20 | 10 |  | 395 |

AH = all healthy feet from sheep where all four feet were healthy; HD = Healthy feet from sheep where one or more feet had signs of footrot; ID = Interdigital dermatitis; SFR = Severe footrot; *Flocks that were *aprB2* positive; ^a^ number of foot swabs collected per flock; ^b^ number of foot swabs pooled per flock; **^c^** number of foot swabs by foot phenotype; **^c^** number of foot pooled samples by foot phenotype.
