# Supplementary material for: Impact of Strain Variation of Dichelobacter nodosus on Disease Severity and Presence in Sheep Flocks in England
Source: Front Vet Sci. 2021 Aug 16;8:713927. doi: 10.3389/fvets.2021.713927 (PMC8415419; doi:10.3389/fvets.2021.713927)
Supplement: Supplementary file 2 [file Table_2.DOCX]

Supplementary Table 2. Distribution of 298 pooled samples positive for at least one serogroup by serogroups A-I by foot phenotype, 395 samples from 3 visits to 24 flocks in England with clinical footrot.

| Foot phenotype Serogroup | | | | | | |  |  | | | |
| --- | --- | --- | --- | --- | --- | --- | --- | --- | --- | --- | --- |
|  | A | B | C | D | E | F | G | H | I | Total |  |
| AH | 2 | 4 | 3 | 0 | 1 | 0 | 0 | 5 | 0 | 15 |  |
| HD | 6 | 16 | 7 | 6 | 6 | 1 | 0 | 23 | 5 | 70 |  |
| ID1 | 8 | 15 | 6 | 4 | 4 | 1 | 1 | 19 | 3 | 61 |  |
| ID2 | 19 | 21 | 4 | 9 | 9 | 0 | 3 | 32 | 7 | 104 |  |
| ID3 | 12 | 21 | 6 | 4 | 4 | 2 | 1 | 23 | 3 | 76 |  |
| ID4 | 8 | 9 | 3 | 3 | 2 | 0 | 1 | 12 | 0 | 38 |  |
| SFR1 | 14 | 16 | 7 | 4 | 4 | 1 | 3 | 23 | 5 | 77 |  |
| SFR2 | 4 | 7 | 3 | 2 | 2 | 0 | 0 | 6 | 2 | 26 |  |
| SFR3 | 4 | 8 | 1 | 0 | 4 | 0 | 0 | 6 | 0 | 23 |  |
| SFR4 | 2 | 2 | 1 | 0 | 1 | 0 | 0 | 3 | 0 | 9 |  |
| Total | 79 | 119 | 41 | 32 | 37 | 5 | 9 | 152 | 25 | 499 |  |

Foot phenotypes are AH = healthy feet from sheep where all four feet healthy; HD = healthy feet from sheep where one or more feet had footrot; ID = Interdigital dermatitis; SFR = severe footrot: 1, 2, 3, 4 = severity score of ID or SFR.
