# Supplementary material for: Impact of Strain Variation of Dichelobacter nodosus on Disease Severity and Presence in Sheep Flocks in England
Source: Front Vet Sci. 2021 Aug 16;8:713927. doi: 10.3389/fvets.2021.713927 (PMC8415419; doi:10.3389/fvets.2021.713927)
Supplement: Supplementary file 3 [file Table_3.DOCX]

Supplementary Table 3. Number and percentage of samples positive for serogroups A-I for 24 flocks in England with clinical footrot by visit (1-3), by flock (all 24), by all pooled samples (395) and by single swab samples (53).

| Serogroup | A | B | C | D | E | F | G | H | I |
| --- | --- | --- | --- | --- | --- | --- | --- | --- | --- |
| Visit 1, 24 flocks |  |  |  |  |  |  |  |  |  |
| N^o^. | 13 | 17 | 6 | 6 | 3 | 3 | 2 | 17 | 5 |
| % | 54.2 | 70.8 | 25 | 25 | 12.5 | 12.5 | 8.3 | 70.8 | 20.8 |
| Visit 2, 24 flocks |  |  |  |  |  |  |  |  |  |
| N^o^. | 12 | 17 | 8 | 6 | 8 | 1 | 2 | 17 | 5 |
| % | 50.0 | 70.8 | 25 | 25 | 33.3 | 4.2 | 8.3 | 70.8 | 20.8 |
| Visit 3, 21 flocks |  |  |  |  |  |  |  |  |  |
| N^o^. | 9 | 9 | 7 | 3 | 5 | 1 | 2 | 15 | 4 |
| % | 37.5 | 37.5 | 29.2 | 12.5 | 20.8 | 4.2 | 8.3 | 62.5 | 16.7 |
| All 24 flocks |  |  |  |  |  |  |  |  |  |
| N^o^. | 12 | 21 | 13 | 9 | 9 | 4 | 4 | 23 | 8 |
| % | 50.0 | 87.5 | 54.2 | 37.5 | 37.5 | 16.7 | 16.7 | 95.8 | 33.3 |
| All 395 swab samples |  |  |  |  |  |  |  |  |  |
| N^o^. | 79 | 119 | 41 | 32 | 37 | 5 | 9 | 152 | 25 |
| % | 20.1 | 30.2 | 10.4 | 8.1 | 9.4 | 1.3 | 2.3 | 38.6 | 6.3 |
| 53 single swab samples |  |  |  |  |  |  |  |  |  |
| N^o^. | 9 | 15 | 6 | 3 | 6 | 1 | 2 | 21 | 3 |
| % | 17.0 | 28.3 | 11.3 | 5.7 | 11.3 | 1.9 | 3.8 | 39.6 | 5.7 |

No. = number positive; % = percentage positive.
