# Supplementary material for: Impact of Strain Variation of Dichelobacter nodosus on Disease Severity and Presence in Sheep Flocks in England
Source: Front Vet Sci. 2021 Aug 16;8:713927. doi: 10.3389/fvets.2021.713927 (PMC8415419; doi:10.3389/fvets.2021.713927)
Supplement: Supplementary file 4 [file Table_4.DOCX]

Supplementary Table 4. Number of serogroups (A-I) detected per visit per flock for three visits (1-3) to 24 flocks in England with clinical footrot.

| Flock | Number of serogroups detected | | | Serogroups detected across three visits |
| --- | --- | --- | --- | --- |
|  | *Visit 1* | *Visit 2* | *Visit 3* |  |
| 1 | 0 | 5 | 5 | A; B; C; D; E; H |
| 2 | 5 | 3 | 0 | A; B; D; F; H; I |
| 3 | 2 | 0 | 1 | B; C; H |
| 4 | 6 | 5 | 0 | B; C; D; E; H; I |
| 5 | 3 | 3 | 5 | B; C; E; F; H; I |
| 6 | 2 | 4 | 4 | B; C; D; G; H; I |
| 7 | 3 | 1 | M | B; H; I |
| 8 | 3 | 5 | 5 | A; B; C; E; H |
| 9 | 3 | 0 | 1 | A; F; H |
| 10 | 2 | 3 | 3 | A; B; H |
| 11 | 4 | 1 | 1 | A; B; C; H |
| 12 | 3 | 4 | 1 | A; B; C; E; F; H |
| 13 | 2 | 4 | 1 | B; C; G; H |
| 14 | 3 | 3 | 0 | B; G; H |
| 15 | 3 | 3 | 5 | A; B; D; E; G; H |
| 16 | 5 | 6 | 2 | A; B; C; D; E; H |
| 17 | 5 | 6 | 4 | A; B; C; D; H; I |
| 18 | 4 | 6 | 3 | A; B; C; D; E; H; I |
| 19 | 4 | 4 | 5 | A; B; D; E; H; I |
| 20 | 3 | 3 | 3 | A; B; H |
| 21 | 3 | 5 | 3 | A; B; C; F; H |
| 22 | 0 | 0 | 0 | 0 |
| 23 | 1 | 2 | 2 | A; H |
| 24 | 3 | 0 | 2 | A; B; H |

M = missing, swab samples were not collected.
