# Supplementary material for: Impact of Strain Variation of Dichelobacter nodosus on Disease Severity and Presence in Sheep Flocks in England
Source: Front Vet Sci. 2021 Aug 16;8:713927. doi: 10.3389/fvets.2021.713927 (PMC8415419; doi:10.3389/fvets.2021.713927)
Supplement: Supplementary file 5 [file Table_5.DOCX]

Supplementary Table 5. Probability of detecting *D. nodosus* by disease severity (*D*) used in the simulation to investigate the probability that all serogroups were detected.

| Disease state | $\boldsymbol{D}_{\boldsymbol{State}}$ | 95% credible intervals |
| --- | --- | --- |
| AH | 0.105 | 0.09 – 0.11 |
| HD | 0.126 | 0.12 – 0.13 |
| ID1 | 0.184 | 0.17 – 0.19 |
| ID2 | 0.429 | 0.40 – 0.44 |
| ID3 | 0.661 | 0.62 – 0.68 |
| ID4 | 0.792 | 0.73 – 0.85 |
| FR1 | 1.0 | Default |
| FR2 | 0.588 | 0.55 – 0.62 |
| FR3 | 0.863 | 0.80 – 0.88 |
| FR4 | 0.621 | 0.51 – 0.67 |
